# Limited global awareness of invasive alien species and little support for their management

**DOI:** 10.1016/j.isci.2026.117811

**Published:** 2026-10-08

**Authors:** Ana Novoa, Ross Shackleton, Sven Bacher, Pavel Pipek, Ratcha Chaichana, Ali El-Keblawy, Bella Galil, Margherita Gioria, Marina Golivets, Tina D'Hertefeldt, Makihiko Ikegami, Ivan Jarić, Christian A. Kull, Nitya P. Mohanty, Zarah Pattison, Aníbal Pauchard, Jan Pergl, Cristina Preda, Klára Pyšková, Jonatan Rodríguez, Kateřina Štajerová, Thi Hai van Nguyen, Ayşe Yazlık, Rafael D. Zenni, Petr Pyšek

**Affiliations:** 1 Department of Invasion Ecology, Institute of Botany, Czech Academy of Sciences, CZ-25243 Průhonice, Czech Republic; 2 Estación Experimental de Zonas Áridas, Consejo Superior de Investigaciones Científícas (EEZA-CSIC), Almería, Spain; 3 Swiss Federal Institute for Forest, Snow and Landscape Research, WSL, Zürcherstrasse 111, 8903 Birmensdorf, Switzerland; 4 Department of Botany and Zoology, Centre for Invasion Biology, Stellenbosch University, Stellenbosch, South Africa; 5 Institute of Geography and Sustainability, University of Lausanne, Lausanne, Switzerland; 6 Department of Biology, University of Fribourg, 1700 Fribourg, Switzerland; 7 Department of Ecology, Faculty of Science, Charles University, Viničná 7, 12844 Prague, Czech Republic; 8 Department of Environmental Technology and Management, Faculty of Environment, Kasetsart University, Bangkok 10900, Thailand; 9 Department of Applied Biology, College of Sciences, University of Sharjah, PO Box Sharjah 27272, UAE; 10 Steinhardt Museum of Natural History, Israel National Center for Biodiversity Studies, Tel Aviv University, Tel Aviv, Israel; 11 Department of Biology, University of Pisa, Via Derna 1, 56126 Pisa, Italy; 12 Helmholtz Centre for Environmental Research – UFZ, Halle, Germany; 13 Halmstad University, Halmstad, Sweden; 14 Lake Biwa Branch Office, National Institute for Environmental Studies, Otsu, Shiga, Japan; 15 Université Paris-Saclay, CNRS, AgroParisTech, Ecologie Systématique Evolution, 12 Rue 128, 91190 Gif-sur-Yvette, France; 16 Biology Centre of the Czech Academy of Sciences, Institute of Hydrobiology, Na Sádkách 702/7, 370 05 České Budějovice, Czech Republic; 17 Centre for Ecological Sciences, Indian Institute of Science, Bangalore, Karnataka 560 012, India; 18 Département Adaptations du Vivant, UMR 7179 C.N.R.S/M.N.H.N, Bâtiment d’Anatomie Comparée, 75005 Paris, France; 19 Biological and Environmental Sciences, University of Stirling, Stirling FK9 4LA, UK; 20 Laboratorio de Invasiones Biológicas (LIB), Facultad de Ciencias Forestales, Universidad de Concepción, Concepción, Chile; 21 Institute of Ecology and Biodiversity (IEB), Concepción, Chile; 22 Department of Natural Sciences, Ovidius University of Constanta, Universitatii Alley, Building B, 900470 Constanta, Romania; 23 CRETUS, Department of Functional Biology, Faculty of Biology, Universidade de Santiago de Compostela, 15782 Santiago de Compostela, Spain; 24 Department of Plant Protection, Faculty of Agriculture, Düzce University, Düzce, Türkiye; 25 Departamento de Ecologia e Conservação, Instituto de Ciências Naturais, Universidade Federal de Lavras, Lavras, Brazil; 26 Wyss Academy for Nature, University of Bern, Bern, Switzerland

**Keywords:** alien species, biological invasions, exotic species, public knowledge, public perceptions, public survey

## Abstract

Invasive alien species (IAS) are one of the main drivers of global change and can cause a wide range of negative ecological and social impacts. Addressing these impacts requires management actions, which can fail to be effective when not supported by the public. Here, we conducted the first global survey on people’s awareness and perceptions of IAS and support for their management. Our results revealed low public awareness and understanding of biological invasions, and suggest that these are mainly influenced by people’s sociodemographic status rather than the social-ecological contexts. Greater awareness of IAS was associated with more negative perceptions toward them and greater support for their management. A large majority of people did not support the use of most management measures. These results suggest that limited public awareness may represent a major obstacle to successfully managing IAS globally, highlighting the need for broader public communication, outreach, and engagement.

## Highlights

• A global survey reveals limited public awareness of invasive alien species
• Socio-demographic factors affect awareness of invasive species more than socio-ecological contexts
• Greater awareness is linked to more negative perceptions and greater management support
• Overall, most people globally do not support the management of invasive alien species

## Graphical abstract

**Figure:**
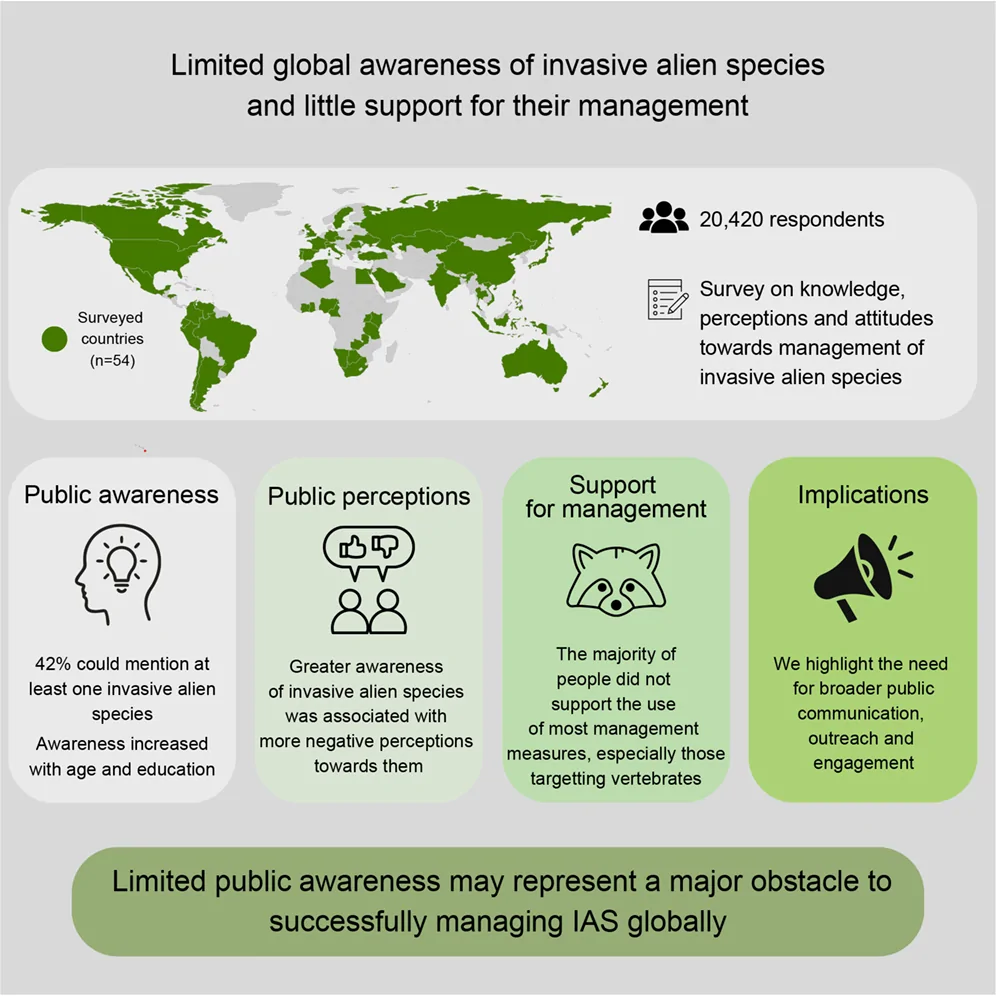

## Teaser

nature conservation; biological sciences; social sciences

## Introduction

Humans have intentionally and/or unintentionally introduced species to regions outside their native ranges for centuries.^1^^,^^2^^,^^3^^,^^4^ Between 6% and 23% of introduced alien species become invasive, causing a wide range of negative ecological and social impacts.^5^ These impacts include altering biodiversity and ecosystem processes^6^^,^^7^ and harming economies, local livelihoods, and human well-being.^8^^,^^9^^,^^10^ Consequently, management actions aiming to reduce the impacts of invasive alien species (IAS) have been implemented in many parts of the world.^11^^,^^12^^,^^13^

Achieving workable management strategies for IAS depends, to a large extent, on public support.^14^^,^^15^^,^^16^^,^^17^^,^^18^ While conservation practitioners and scientists are generally aware of the negative impacts of IAS and advocate for their control, their management can be challenged by public opposition. For example, the management of invasive alien mammals is often perceived by the public as cruel and immoral (i.e., the notion that any creature has the right to live^19^^,^^20^). IAS can also provide economic benefits for some stakeholders^21^^,^^22^^,^^23^ or be perceived as charismatic and having intrinsic value.^24^ As such, understanding public awareness and perceptions of IAS can help decision-making in situations of conflict and opposition to their management,^18^^,^^25^ and help develop acceptable management approaches to ensure social-ecological benefits.

However, understanding and generalizing what people know about and how they perceive IAS often depends on the local social-ecological contexts.^25^ Several studies have assessed public awareness and perceptions of IAS, but only locally and within specific stakeholder groups and the results show substantial variation^26^^,^^27^^,^^28^^,^^29^^,^^30^^,^^31^^,^^32^^,^^33^^,^^34^; therefore, broad-scale generalizations cannot be inferred. Studies that consider public awareness and perceptions across large scales and broad sociocultural contexts are still lacking.

Here, we have addressed this knowledge gap by assessing and comparing public awareness and perceptions of IAS and how these influence people’s support for their management in a global survey, spanning 54 countries, across all inhabited continents. We also investigated the major social-ecological drivers that affect people’s awareness, perceptions, and attitudes toward management. To accomplish this, we conducted a series of standardized nationally balanced surveys in terms of age (between 18 and 75 years old), gender, and location (urban and rural areas), in 23 languages to gauge public awareness and perceptions of IAS and their management with 22,178 respondents globally (for details regarding the data collection, see the STAR Methods). These data provided a unique insight into how public awareness of IAS globally influences people’s perceptions toward IAS across taxonomic groups and support for their management.

## Results

### Global awareness of IAS

First, we assessed public awareness of IAS by asking respondents whether they could name any IAS occurring in their country (see STAR Methods). Overall, 41.6% of respondents could mention at least one IAS. Education (χ^2^ = 87.48, df = 3, *p* < 0.001) and age (χ^2^ = 66.90, df = 2, *p* < 0.001) showed the strongest influence on respondents’ awareness. Older people had a better knowledge of IAS than younger people. People with a university degree had a better knowledge of IAS than those with secondary education, and the latter showed more awareness than those with primary education. The size of the locality of residence (χ^2^ = 9.53, df = 3, *p* = 0.023) was also associated with awareness. People living in big cities showed higher awareness of IAS than people living in medium or small cities, or in rural areas (Figure 1A). Independent of this, the number of alien species recorded in the country (χ^2^ = 6.06, df = 1, *p* = 0.014) was also positively associated with awareness.

**Figure 1:**
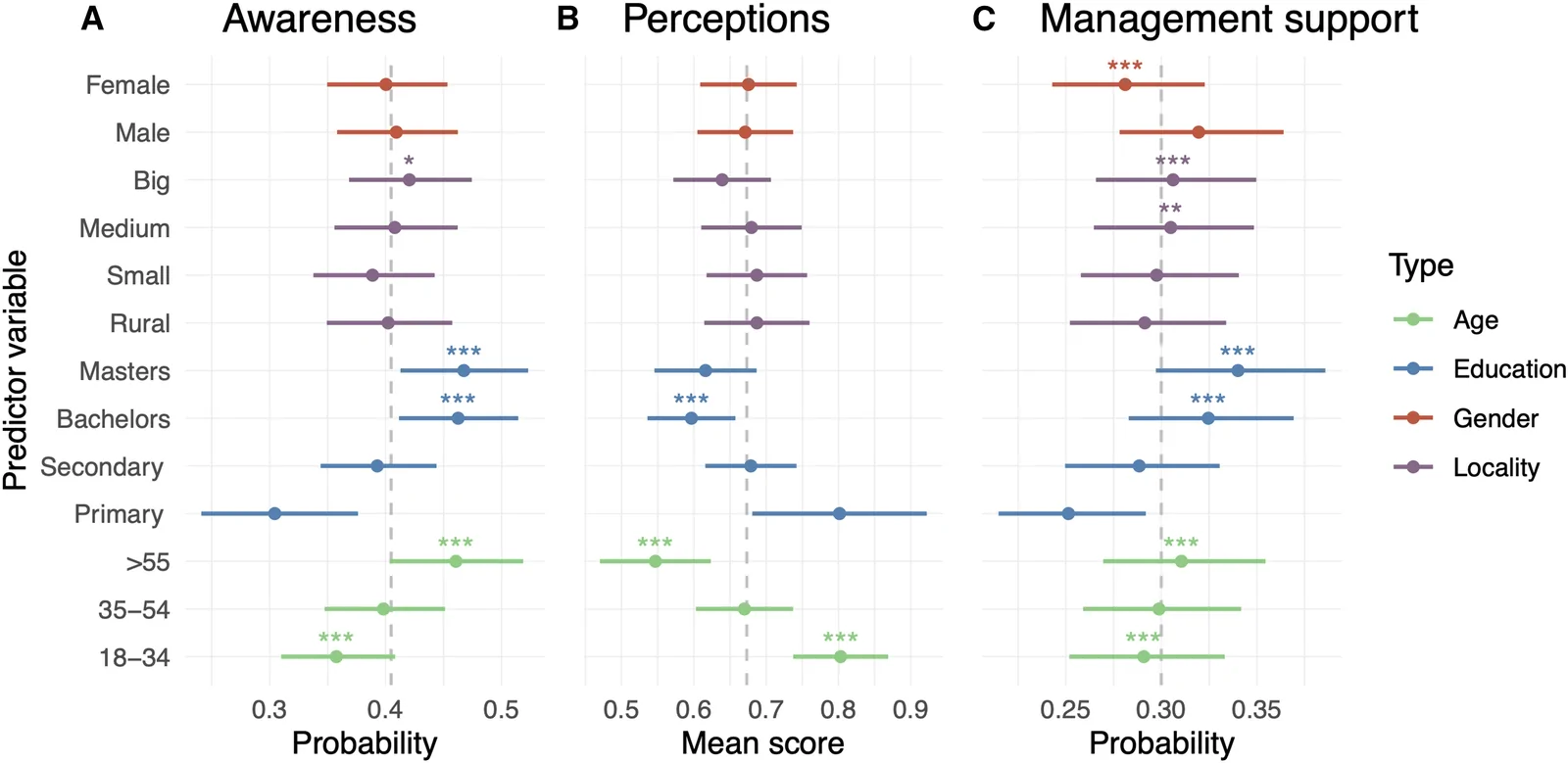
Influence of socio-demographic aspects on respondents’ awareness, perceptions, and support for management Marginal effects of age, education level, gender, and locality size on respondents’ (A) awareness (i.e., whether respondents could name at least one IAS occurring in their country) and (B) perceptions (i.e., whether respondents rated the first three words that came to their mind when they thought about IAS as negative, neutral, or positive, with score increasing in this order) and (C) management support (i.e., whether respondents indicated that they would support the management of IAS). Circles and horizontal lines indicate the effects’ means and 95% CIs. Vertical gray lines show the overall marginal effect. Significant pairwise differences from the baseline groups are indicated with ∗*p* < 0.05; ∗∗*p* < 0.01; ∗∗∗*p* < 0.001. Baseline groups were 35–54 (age), secondary (education), male (gender), small (locality).

A total of 225 different invasive alien taxa were mentioned by respondents (Figure 2). Invasive alien vertebrate species were mentioned most often (Figure 2A), particularly mammals and fish (Figure 2D). For plants, respondents mainly mentioned trees and herbs (Figure 2B), and insects were most commonly mentioned within the category of invertebrates (Figure 2C).

**Figure 2:**
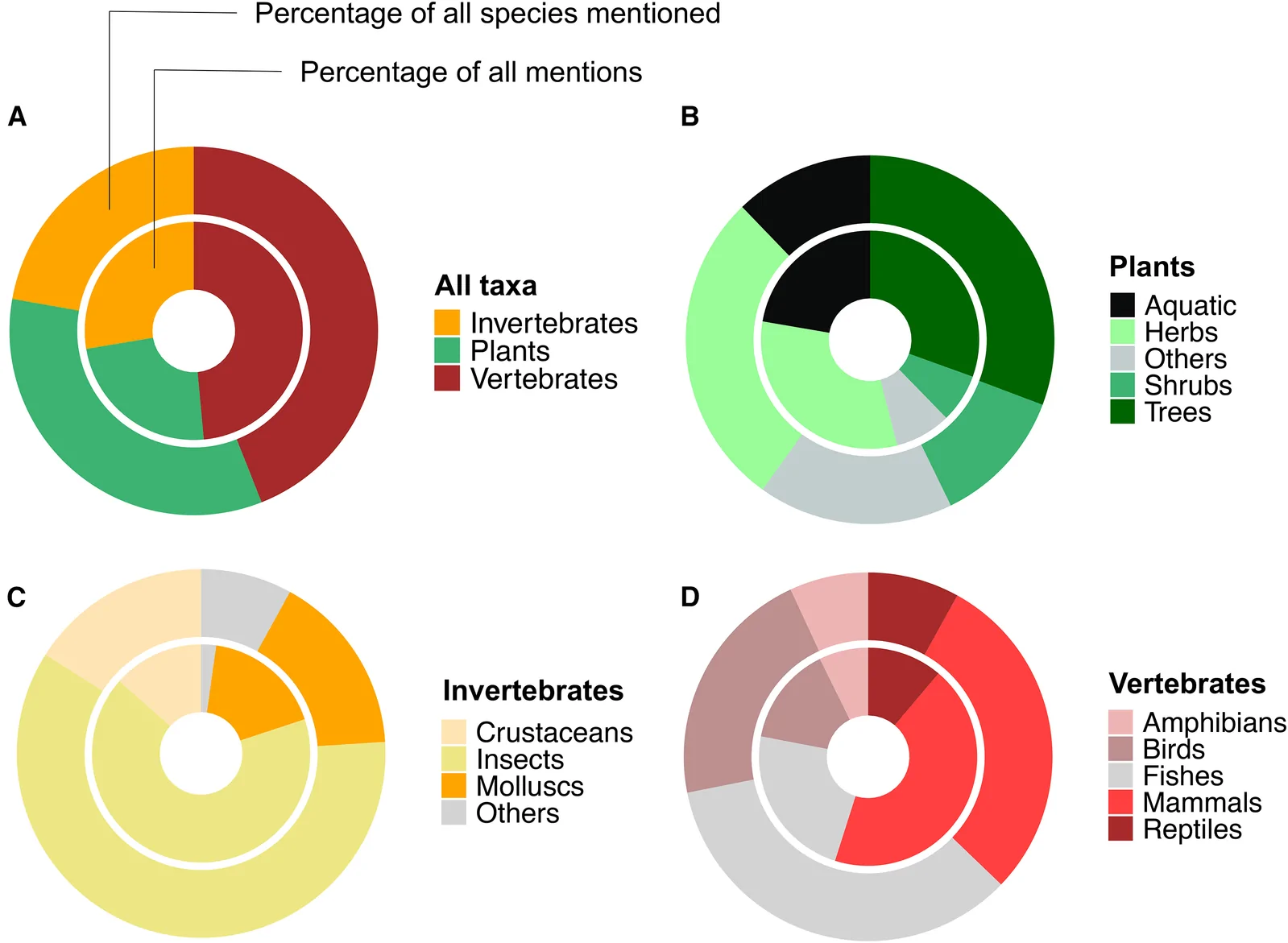
Invasive alien species mentioned by respondents Percentage of the IAS mentioned by respondents (outer circles) and of all mentions (inner circles) of (A) all taxa, (B) plants (76 species, mentioned by 3,272 respondents), (C) invertebrates (50 species, mentioned by 3,800 respondents) and (D) vertebrates (99 species, mentioned by 6,668 respondents).

Across all countries surveyed, the three most often mentioned taxa were invasive alien mosquitoes (*Aedes* spp.), mentioned by 803 respondents; rats (*Rattus* spp.), by 626 respondents; and water hyacinth (*Pontederia crassipes*), by 545 respondents (Figure 3).

**Figure 3:**
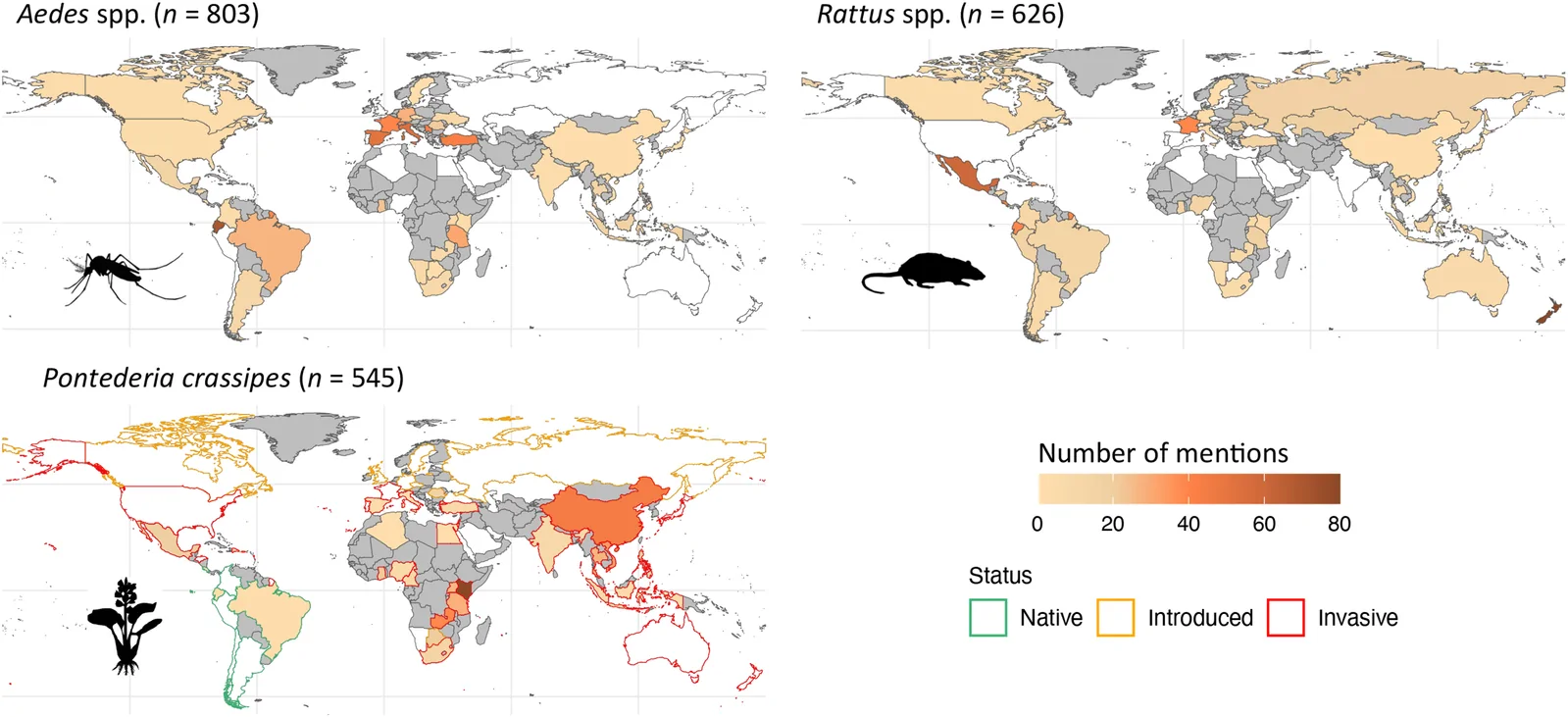
Number of respondents per country mentioning the most mentioned taxa globally Countries shaded in gray are those in which the survey was not distributed, and therefore no data are available. For *Pontederia crassipes*, the borders of the surveyed countries are colored according to the status of the species (i.e., native, introduced, invasive) in each country. This was not done for *Aedes* spp. and *Rattus* spp., as these taxa include several species with different native and introduced ranges. Silhouettes of *Aedes* spp. and *Rattus* spp. were obtained from PhyloPic (https://phylopic.org/) and both are in the public domain. The silhouette of *Pontederia crassipes* was created by James Hardwick.

### Global perceptions of IAS

To assess perceptions, we asked respondents to provide the first three words that immediately came to their mind when they thought about IAS, and to score them on a scale from very negative, negative, neutral, positive to very positive. Similar to our findings on public awareness, education (χ^2^ = 34.58, df = 3, *p* < 0.001) and age (χ^2^ = 128.35, df = 2, *p* < 0.001) had the strongest influence on respondents’ perceptions of IAS. People with a university degree had more negative perceptions toward IAS than those with secondary education, and the latter showed more negative perceptions than people with primary education only. Regarding age, older people had significantly more negative perceptions of IAS than younger people. The size of the locality of residence (χ^2^ = 8.41, df = 3, *p* = 0.038) was also associated with perceptions, where people living in big cities showed more negative perceptions toward IAS (Figure 1B).

Accordingly, after controlling for socio-demographic and socio-ecological predictors, respondents who could name at least one IAS present in their country were significantly more likely to perceive IAS negatively (ordinal regression, *p* < 0.001) (Figure 4).

**Figure 4:**
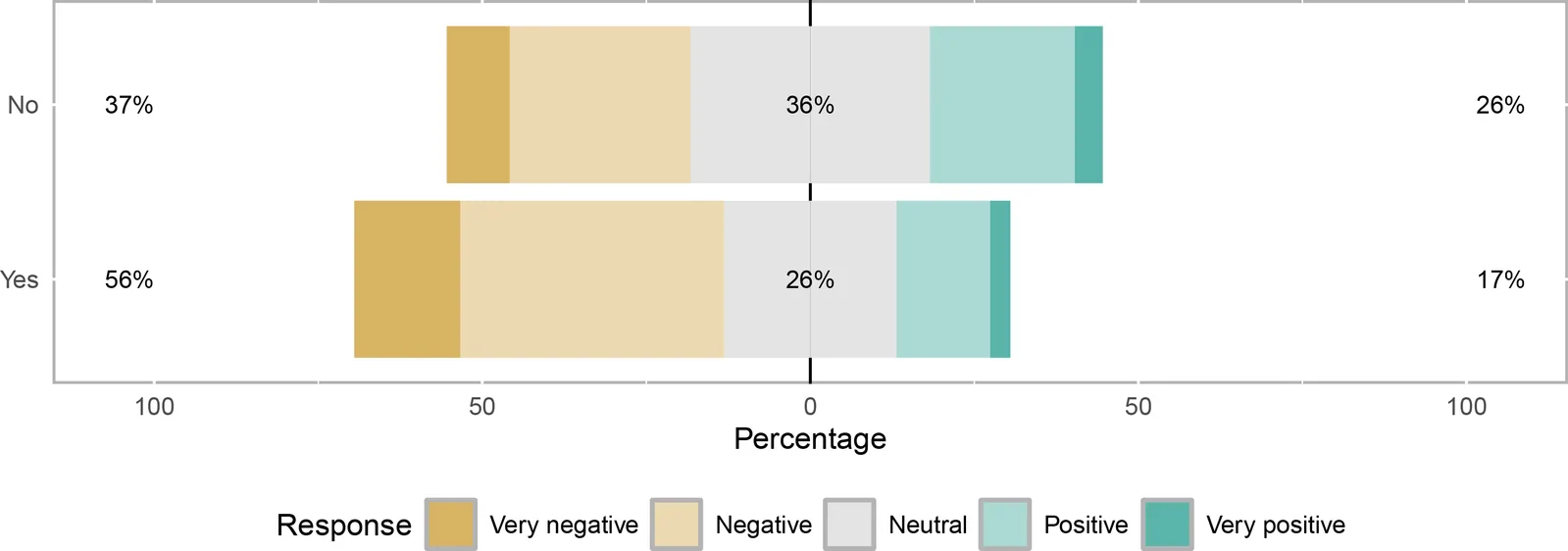
Survey respondents’ perceptions of IAS according to their awareness of IAS, i.e., whether respondents could (yes) or could not (no) name at least one IAS occurring in their country Percentages indicate the respondents that scored the first three words that immediately came to their mind when they thought about IAS as very negative (dark brown shading and to the left), negative (light brown shading), neutral (gray, center), positive (light green shading) and very positive (dark green shading and to the right).

### Lack of support for IAS management

Our results suggest that, globally, most individual management actions targeting IAS were supported by fewer than half of respondents (Figure 5). Moreover, management actions targeting invasive alien vertebrates were significantly less supported by respondents than those targeting invasive alien plants (*β* = −0.451, *p* = 0.001) or invasive alien insects (*β* = −0.581, *p* = 0.011).

**Figure 5:**
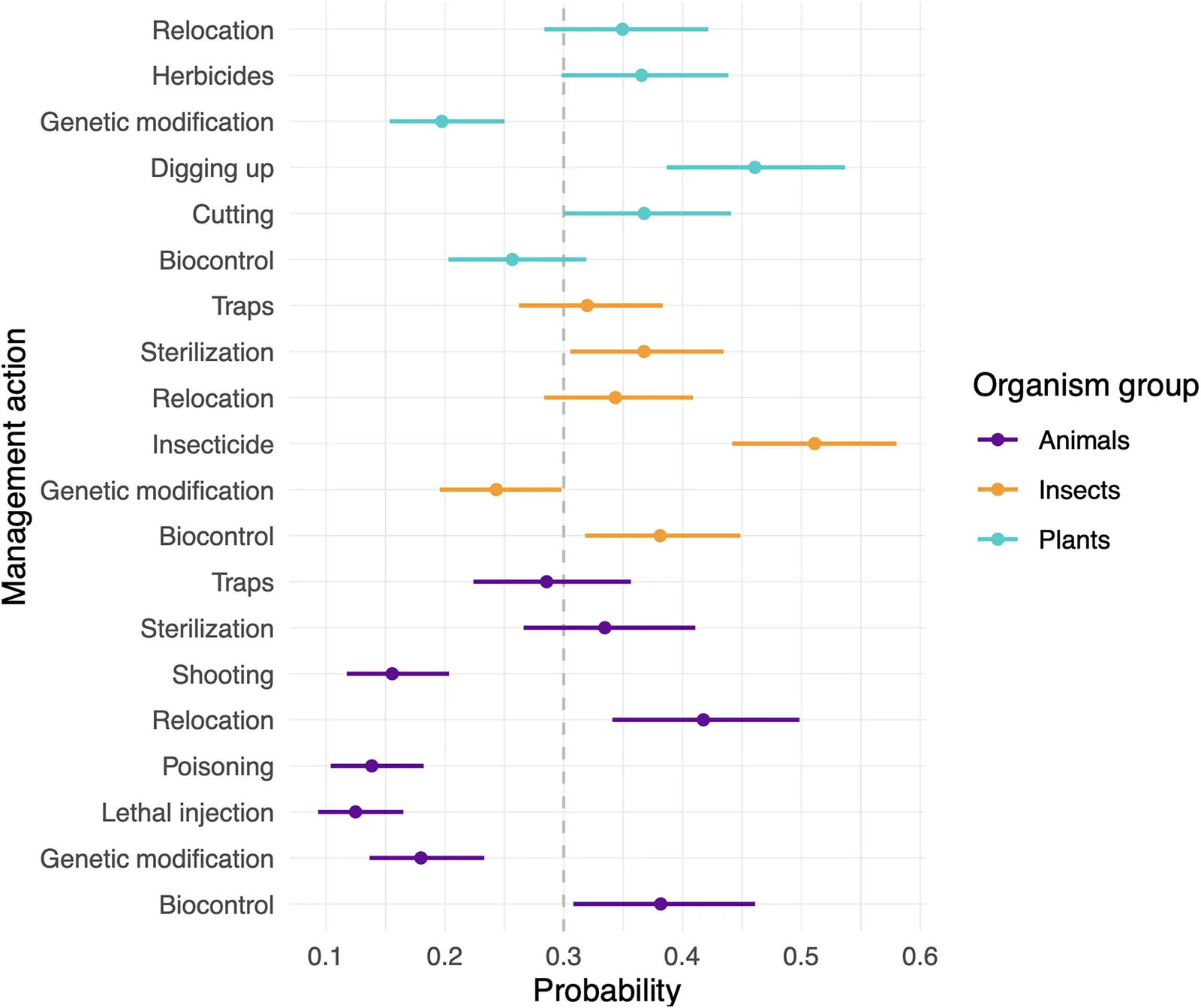
Average support of respondents for individual management actions Vertical dotted gray line indicates the overall average predicted support across all respondents and actions. Circles and horizontal lines indicate the effects’ means and 95% CIs, respectively. Most actions were supported by fewer than half of the respondents.

Similar to our findings on public awareness and perceptions of IAS, support for management was influenced by age (older people are more supportive toward management actions than younger people), level of education (people with a university degree showed more support for management than people with secondary and primary education) and the size of the locality of residence (people living in big or medium cities supported more management actions than people living in small cities or rural areas) (Figure 1C). Finally, female respondents showed less support for management actions than male respondents.

After controlling for socio-demographic and socio-ecological predictors, awareness of IAS and more negative perceptions toward IAS were positively associated with support for management actions (Table S1).

## Discussion

Although our results indicate that a substantial proportion of respondents were familiar with at least one IAS, awareness appeared lower than that reported for other major environmental issues, such as climate change, which have received considerably greater public attention and media coverage globally. For example, Lee et al.^35^ showed public awareness levels of climate change to be approximately 64% globally and the Pew Research Center’s global annual surveys found that a median of 75% of respondents across 19 countries globally label climate change as a major threat.^36^

Mosquitoes (*Aedes* spp.), rats (*Rattus* spp.), and water hyacinth (*Pontederia crassipes*) were the three most mentioned IAS across all continents surveyed. Representatives of all three taxa are listed among the 100 worst IAS globally.^37^ Moreover, mosquitoes and rats are listed as the two invasive alien taxa with the highest economic costs globally,^38^ while water hyacinth is extremely widespread outside its native range,^1^ and causes large-scale social and ecological changes wherever it invades.^39^ Rats and water hyacinth have also caused multiple local extinctions of native species and mosquitoes pose major threats to human health, primarily through the spread of diseases.^40^^,^^41^^,^^42^

In agreement with previous findings of local case studies,^30^^,^^43^ our results suggest that public awareness of IAS is more driven by the socio-demographic status of individuals than by the social-ecological contexts they live in.^25^ Similarly, education, age, and size of the locality of residence influenced respondents’ perceptions of IAS and support for management. The strong similarities in demographic factors affecting people’s awareness, perceptions, and support for management can be explained by the former influencing the latter.^44^^,^^45^

Most people showed little or no support for any management action targeting IAS. Only the use of insecticides was deemed acceptable for the majority of respondents, while actions aimed at genetically modifying IAS (from any taxonomic group) to reduce their invasion risk were not supported by a great majority of respondents. Accordingly, genetically modifying IAS still remains controversial among academics and managers.^46^ Also, in agreement with the results of previous local studies, management of invasive alien plants or insects was generally more acceptable for the public than that of mammals.^14^^,^^47^ One possible explanation is that some invasive vertebrates, particularly mammals, may elicit greater public sympathy because of their perceived charisma or cultural value.^24^^,^^31^

We also observed differences in risk perception (i.e., the perception that there is a risk of something going wrong if a management action is implemented) between men and women (women tend to be, on an average, more risk averse; see Xiao and McCright^48^ and references therein). In general, men have lower risk perception concerning management actions (referred to as the “white male effect”^49^), including those targeting IAS.^14^^,^^50^^,^^51^

### Limitations of the study

This study provides a large-scale view of public awareness of IAS, yet several limitations should be acknowledged. First, awareness was assessed by asking respondents to list at least one IAS occurring in their country. Although this offers a consistent measure across countries, it reflects only one dimension of knowledge and may not capture experiential, practical, or locally grounded understanding.

Second, distributing the survey through SurveyMonkey restricts participation to individuals with internet access, which may skew samples toward more connected populations, particularly in countries where internet access is uneven. Although we balanced samples by age, gender, and urban/rural residence, other aspects of representativeness (e.g., media exposure, occupation, and contact with nature) remain uncertain.

Third, the data represent a single point in time. Consequently, the study cannot assess the influence of awareness campaigns, policy interventions, or changes in species distributions on public awareness, perceptions, and attitudes toward IAS. Repeated cross-sectional surveys would be needed to evaluate how these evolve.

Taken together, these considerations highlight the need for more inclusive sampling strategies, broader measures of knowledge, and temporal monitoring to deepen understanding of public awareness, perceptions, and attitudes toward IAS.

### Societal and policy implications

Despite the discussed limitations, our survey yielded strong evidence that the majority of people globally were unable to name an IAS in their country, do not perceive them as problematic, and most individual management measures were supported by fewer than half of respondents. This is substantially less than awareness of other environmental issues, namely climate change. This is very worrisome given the difficulties in regulating climate change and the fact that the threat from IAS is considered to be of a similar magnitude.^4^^,^^52^ Such a general lack of awareness of the problem and support for the control of IAS is likely to add to the further spread and major social-ecological impacts of biological invasions, which are predicted to increase substantially in the future,^53^ especially when biological invasions are acting in synergy with other major global change factors like climate and land-use change.^54^

Multiple factors were theorized to affect people’s awareness and perceptions of IAS and support for their management,^25^ and in this study, we show that, in general, those relating to the individual (e.g., age, gender, and education) were better predictors of variation in awareness and perceptions than broader social and ecological contextual factors (e.g., prevailing land use in the country of residence that is reflected in people’s occupation and lifestyle). However, differences among countries in IAS-related legislation, policy frameworks, and public communication efforts may also contribute to variation in public awareness, perceptions, and support for management.^25^ Further, building on the findings of local case studies,^31^ our results show that globally, people who are aware of IAS more often perceive them as negative and support management measures. Raising awareness through education has been shown to effectively increase support of IAS management by the public,^31^^,^^55^ but the development of best practices to do this is needed.^55^ Integrating IAS into school curricula, media, environmental education programmes, and professional training in sectors linked to introduction pathways (e.g., agriculture, aquaculture, forestry, and tourism) could help improve long-term public awareness and support for management.^56^ In addition, better understanding and incorporating indigenous views and local knowledge (which might be different from scientific and “Western” ones) needs to be considered increasingly with regard to IAS policy and co-management. This could mean that we may need to increasingly acknowledge the more nuanced cultural perspectives of IAS rather than them just being “good” or “bad.”^4^^,^^57^^,^^58^^,^^59^ Research on the social dimensions is increasing but still lags far behind ecologically focused work.^60^ We suggest that such studies should be promoted to improve management, minimize the negative impacts of biological invasions globally, and guide policy and management.

## Resource availability

### Lead contact

Further information and requests for resources and data should be directed to the lead contact, Ana Novoa (email: novoa.perez.ana@gmail.com).

### Materials availability

This study did not generate new materials.

### Data and code availability

• All data supporting the findings of this study are deposited in Dryad (https://doi.org/10.5061/dryad.sf7m0cgpn). The dataset includes anonymized survey responses and metadata, following FAIR data principles. Any additional information is available from the corresponding author upon reasonable request.
• This paper does not report original code.

## Acknowledgments

A.N., J.P., K.P., K.Š., and P. Pipek acknowledge funding from EXPRO grant no. 19-28807X (Czech Science Foundation), which supported data collection for this paper. P. Pyšek acknowledges grant no. 26-21350S (Czech Science Foundation), which supported finalization of the paper. A.N., J.P., K.P., K.Š., P. Pipek, and P. Pyšek also acknowledge support from long-term research development project RVO 67985939 (Czech Academy of Sciences). A.N. was supported by the MCIN/AEI/10.13039/501100011033 and the FSE+ (grant no. RYC2022-037905-I). R.S. acknowledges funding from the University of Lausanne through the Matterhorn funding scheme which contributed to data collection for this paper. S.B. acknowledges funding from the Swiss National Science Foundation (grant no. 31003A_179491 and 31BD30_184114) and the University of Fribourg, which contributed to data collection for this paper. N.P.M. was supported by the Indian Institute of Science’s Raman postdoctoral fellowship and would like to thank Sachin Vaishampayam for help with translations. J.R. acknowledges funding from the Spanish Ministry of Universities under application 33.50.460A.752 and the European Union NextGenerationEU/PRTR through a contract Margarita Salas of the Universidade de Vigo (UP2021-046). We thank Vasco Ferreira Da Costa, Rahila Junika, John R. Mbwambo, Desika Moodley, Lisanna Schmidt and Qiang Yang for help with data preparation and/or translations. A.P. was supported by grant ANID PIA/BASAL FB210006.

## Author contributions

A.N., P. Pyšek, R.S., and S.B. designed the study; A.N., R.S., P. Pipek, J.P., K.P., J.R., K.Š., and P. Pyšek designed the questionnaire; A.N., S.B., R.C., A.E.-K., B.G., M. Gioria, M. Golivets, M.I., I.J., C.A.K., N.P.M., J.P., C.P., T.D.H., T.H.v.N., R.D.Z., and A.Y. translated the questionnaires; A.N., S.B., R.C., A.E.-K., B.G., M. Gioria, M. Golivets, M.I., I.J., C.A.K., N.P.M., J.P., C.P., P. Pipek, K.P., A.P., K.Š., J.R., Z.P. and R.S. screened the species mentioned by respondents; A.N. and M. Golivets analyzed the data. A.N. wrote the paper. All the authors curated the data and approved the final manuscript.

## Declaration of interests

The authors declare no competing interests.

## Declaration of generative AI and AI-assisted technologies in the manuscript preparation process

During the preparation of this manuscript, the authors used ChatGPT (OpenAI) to assist with wording and improve the clarity and readability of the text. After using this tool, the authors reviewed and edited the content as needed and take full responsibility for the content of the published article.

## STAR★Methods

### Key resources table

**Table:** 

REAGENT or RESOURCE	SOURCE	IDENTIFIER
**Deposited data**		

Data used in this study	Dryad Digital Repository	https://doi.org/10.5061/dryad.sf7m0cgpn

**Software and algorithms**		

R version 4.3.2	R Core Team R Foundation for Statistical Computing	https://www.r-project.org

### Experimental model and study participant details

We designed and implemented a questionnaire aiming to recruit approximately 400 adult respondents per country, stratified by age, gender and locality (urban vs. rural). Of all respondents, 51.7% identified as male, 47.6% as female, and 0.8% as other. Regarding age, 58.0% of respondents were aged 18–34 years, 32.1% were aged 35–54 years, and 9.9% were aged 55 years or older.

### Method details

#### Questionnaire

To assess public awareness, perceptions and support for the management of invasive alien species (IAS), we organized a half-day workshop attended by the core authors during which we designed an initial questionnaire. Following the workshop, all authors added, edited or removed the questions included in the initial questionnaire over a two-month period. To evaluate the comprehensibility, clarity, length, and accuracy of the questions, we pilot-tested the questionnaire with 10 respondents.

The final questionnaire (Supplementary file 1) contained three sections aimed at: (1) assessing respondents’ awareness and perceptions of IAS, (2) exploring whether respondents would approve the implementation of different IAS management actions (hereafter support for management), and (3) collecting socio-demographic information from respondents. We avoided directly asking whether respondents were aware of the issue, as such self-assessments may lead to confirmation bias. Similarly, requesting a definition of an invasive alien species (IAS) was considered too complex and potentially off-putting. Instead, in Section 1 of the questionnaire, respondents were asked to name up to five IAS present in their country. We interpreted the ability to name at least one IAS as an indicator of awareness of biological invasions. In this same section, to assess their perceptions, respondents were asked to provide three words that immediately came to their mind when they thought about IAS and to score each word on a scale from −2 (very negative) to +2 (very positive). In section 2, respondents were asked whether they would support (yes/no) 20 different management actions aimed at removing invasive alien insects, vertebrates and plants (Figure 5, Supplementary material). These actions were selected to represent the range of chemical, mechanical and biological measures commonly used in invasive alien species management. Finally, section 3 included four additional questions to gather respondents’ socio-demographic information, including age (18–24 years old, 25–34, 35–44, 45–54, 55–64, 65–74, 75+, or prefer not to say), gender (female, male, prefer not to say or other), education level (no schooling, primary school, secondary school, bachelor’s degree, master’s degree, or other), and approximate size of the locality they live in (rural (<10,000 inhabitants), small (10,001–100,000 inhabitants), medium (100,001–500,000 inhabitants) or big city (>500,000 inhabitants)).

The questionnaires were translated into 23 languages, namely Arabic, Chinese, Czech, English, Estonian, French, German, Hebrew, Hindi, Indonesian, Italian, Japanese, Portuguese, Romanian, Russian, Serbian, Spanish, Swahili, Swedish, Thai, Turkish, Ukrainian and Vietnamese by the co-authors of this study, who are native speakers. Online questionnaires were created in SurveyMonkey (https://surveymonkey.com/).

#### Data collection

Online questionnaires were administered between the 3rd of December 2021 and the 28th of June 2022 (in Russia, questionnaires were administered and data was collected before the 24th of February 2022) by Cint (https://www.cint.com/products/cint-consumer-exchange/panelbook/) in 54 countries, covering all inhabited continents. We obtained a total of 22,178 responses. After data cleaning (removal of incomplete or incoherent responses), the final number of valid responses per country was as follows: Algeria (363), Argentina (388), Australia (377), Botswana (358), Brazil (377), Cameroon (554), Canada (404), Chile (374), China (394), Colombia (369), Costa Rica (432), Czech Republic (384), Dominican Republic (383), Ecuador (399), Egypt (136), Estonia (380), France (441), Germany (385), Ghana (394), Honduras (398), India (293), Indonesia (401), Israel (324), Italy (377), Ivory Coast (389), Japan (342), Kazakhstan (274), Kenya (397), Mexico (368), Namibia (363), New Zealand (374), Nigeria (396), Peru (387), Philippines (383), Portugal (395), Puerto Rico (394), Romania (421), Russia (260), Saudi Arabia (378), Serbia (380), Singapore (405), South Africa (385), Spain (381), Sweden (377), Switzerland (439), Tanzania (389), Thailand (408), Türkiye (388), Uganda (383), Ukraine (388), United Kingdom (390), United States (354), Vietnam (401), and Zambia (346). This resulted in a final sample of 20,420 respondents. All participants provided informed consent and were reimbursed according to their standing agreements with Cint.

#### Data preparation

All answers were translated into English by the co-authors of this study and retained in the analyses. Data analysis and visualization were performed using the R statistical software (v4.0.4.). The IAS mentioned by respondents were screened to assess whether they were indeed IAS in the country of the respondent, using sources such as POWO (https://powo.science.kew.org), GloNAF (https://glonaf.org) or the CABI Compendium (https://www.cabidigitallibrary.org/product/QI). We did not consider any mentioned disease as an IAS. Since respondents could use common or scientific names, all names were then standardized to scientific names. Then, all species were classified according to their taxonomic group as plants (aquatic, herbs, shrubs, trees and other), invertebrates (crustaceans, insects, molluscs and other) and vertebrates (amphibians, birds, fish, mammals and reptiles). To get a proxy for the respondents’ awareness of IAS, all answers were classified as “Yes” or “No”, depending on whether the respondent mentioned at least one IAS occurring in their country or not.

From the World Bank Open Data (https://data.worldbank.org/) portal, we extracted data on the income level status (high, low, lower middle, upper middle) and Gross domestic product (GDP) per capita of each studied country and the percentage of each country’s land that is (a) used by humans (arable, under permanent crops or pastures) and (b) under protection. Additionally, we extracted data on the number of alien species recorded in each studied country from the Global Register of Introduced and Invasive Species (GRIIS).^61^

### Quantification and statistical analyses

As a proxy of the perceptions of respondents toward IAS, the scores given by each respondent to the three words that immediately came to their mind when they thought about IAS were translated into a number (very negative = −2, negative = −1, neutral = 0, positive = 1 and very positive = 2), summed up and divided by three. The final number was classified as very negative (between −1.5 and −2), negative (between −1.5 and −0.5), neutral (between −0.5 and 0.5), positive (between 0.5 and 1.5) or very positive (between 1.5 and 2).

Aiming to identify levels of consensus or disagreement in the perceptions of respondents when thinking about IAS, we used the package ‘agrmt’^62^ to calculate polarization scores in each country. Polarization scores range from 0 to 1, with values closer to 0 indicating increased consensus and values closer to 1 indicating increased disagreement. We then classified polarization scores as low, moderate, and high according to their upper and lower quartiles (i.e., low polarization ≤0.26 and high polarization ≥0.31).

We used generalised mixed-effects models to test the influence of socio-demographic and socio-economic factors on respondents’ awareness, perceptions of biological invasions and support for different IAS management actions. Across all models, respondents’ socio-demographic characteristics (age, education, gender, and locality) and key features of the socio-ecological context of their country (GDP, number of alien species, percentage of land used by humans, and percentage of land protected from human use) were included as fixed effects, with country as a random effect to account for unmeasured variability across countries. We refer to these variables as socio-demographic and socio-ecological “groups” for conceptual clarity, but each variable was included independently in the models. We assessed collinearity among predictors prior to modeling using correlation analyses and Variance Inflation Factors (VIF). No strong correlations were detected among predictors, and all VIF values were below 10, indicating acceptable levels of collinearity. Socio-demographic variables capture individual-level characteristics likely to influence awareness, perceptions, and attitudes toward invasive alien species. Socio-ecological variables reflect broader contextual conditions that may shape people’s experiences and exposure to invasive alien species.

We grouped them conceptually because they represent distinct levels of influence (i.e., individual versus country-level context) rather than combining them. This grouping aids interpretation and comparison across models, while preserving the unique contribution of each variable.

In the models for the management support, we additionally used taxa targeted by the different management actions as a fixed effect, and country and management action as random effects. Awareness and support for management, which were binary (1 = support/aware, 0 = no support/unaware), were modeled using a binomial error distribution with a logit link function with the glmer function of the ‘lme4’ R package.^63^ For respondents’ perceptions, measured on the ordinal scale, we applied an ordinal regression with a probit link function using the clmm function of the ‘ordinal’ R package.^64^ For each categorical variable (i.e., the socio-demographic characteristics of the respondent), we used the category with the median awareness rate (i.e., the median number of respondents able to name an invasive alien species occurring in their country) as the baseline. We calculated marginal fixed effects using the ‘emmeans’ R package^65^ and marginal random effects using the ‘ggeffects’ R package.^66^ For joint tests of the model predictors, we used the joint_effects function, and for pairwise comparisons, the pairs function in the ‘emmeans’ R package.^65^

Finally, to assess whether respondents’ awareness of IAS influenced their perceptions of IAS, and whether respondents’ awareness and perceptions influenced their support for IAS management actions, we fitted additional regression models including awareness and/or perceptions as explanatory variables while controlling for the same socio-demographic and socio-ecological predictors described above.

## Supplemental information

Document S1. Table S1 and questionnaire used in this study
