# Supplementary material for: Limited global awareness of invasive alien species and little support for their management
Source: iScience. 2026 Oct 8;29(11):117811. doi: 10.1016/j.isci.2026.117811 (PMC13677325; doi:10.1016/j.isci.2026.117811)
Supplement: Supplementary file 1 [file mmc1.pdf]

iScience, Volume *29*

Supplemental information

Limited global awareness of invasive alien species and little support for their management

Ana Novoa, Ross Shackleton, Sven Bacher, Pavel Pipek, Ratcha Chaichana, Ali El-Keblawy, Bella Galil, Margherita Gioria, Marina Golivets, Tina D'Hertefeldt, Makihiko Ikegami, Ivan Jarić, Christian A. Kull, Nitya P. Mohanty, Zarah Pattison, Aníbal Pauchard, Jan Pergl, Cristina Preda, Klára Pyšková, Jonatan Rodríguez, Kateřina Štajerová, Thi Hai van Nguyen, Ayşe Yazlık, Rafael D. Zenni, and Petr Pyšek

Table S1 – Results of the generalized linear mixed-effects model testing the influence of respondents' knowledge (i.e., whether respondents could or could not name at least one invasive alien species occurring in their country) and perceptions of invasive alien species (i.e., whether the first three words that immediately came to the mind of respondents when they thought about invasive alien species were negative, neutral or positive) on support management actions (i.e., whether they would or would not support a particular management action), while controlling for socio-demographic and socio-ecological predictors. The model included age, education, gender, locality, GRIIS, percentage of agricultural land, percentage of protected land, and taxa as fixed effects, and country and management action as random effects.

**Predictor**	**Estimate (β)**	**SE**	**z value**	**p value**
Awareness	0.219	0.008	27.71	<0.001
Perceptions	-0.089	0.004	-23.28	<0.001

Document S1. Questionnaire used in this study

Conservation questionnaire

Invasive species are any living thing (an animal, plant, insect, microorganism, etc.) which are brought by people to a new place where they do not naturally occur, and, once they are set free in this new place, survive on their own, reproduce and spread widely.

Which three words/terms spontaneously come to your mind when you think about invasive/alien/introduced/non-native species?

Word 1

Word 2

Word 3

Please, indicate on the scale below how negative or positive you feel about the three words you mentioned in the previous question.

Word 1

Word 2

Word 3

✓
Very negative
Negative
Neutral
Positive
Very positive

Could you name any invasive species occurring in your country (up to five if possible)? Feel free to use common names, local names or scientific names.

Which of the following methods would you support to remove invasive insects? Please, tick all that apply.

- ☐ Insecticides
- ☐ Sterilization
- ☐ Genetic modification
- ☐ Traps
- ☐ Relocation
- ☐ Introducing their natural enemies

Which of the following methods would you support to remove invasive animals, such as mammals, birds, reptiles, amphibians and/or fishes? Please, tick all that apply.

- ☐ Lethal injection
- ☐ Poisoning
- ☐ Shooting
- ☐ Sterilization
- ☐ Traps
- ☐ Relocation
- ☐ Genetic modification
- ☐ Introducing their natural enemies

Which of the following methods would you support to remove invasive plants? Please, tick all that apply.

- ☐ Cutting
- ☐ Digging up
- ☐ Herbicides
- ☐ Introducing their natural enemies
- ☐ Genetic modification
- ☐ Relocation

What is your age?

- ✓
- 18-24 years old
- 25-34 years old
- 35-44 years old
- 45-54 years old
- 55-64 years old
- 65-74 years old
- 75+ years old
- Prefer not to answer

Which gender do you identify as?

- ✓
- Female
- Male
- Prefer not to say
- Other (please specify)

Could you indicate the approximate size of the locality you live in?

- ✓
- Rural (<10 000 inhabitants)
- Small city (10 001 – 100 000 inhabitants)
- Medium city (100 001 – 500 000 inhabitants)
- Big city (> 500 000 inhabitants)
